# Editorial: Pre-clinical Models of PTSD

**DOI:** 10.3389/fnbeh.2019.00237

**Published:** 2019-10-15

**Authors:** Shane A. Perrine, Israel Liberzon

**Affiliations:** ^1^Research Services, John D. Dingell VAMC and Department of Psychiatry and Behavioral Neurosciences, Wayne State University, Detroit, MI, United States; ^2^Department of Psychiatry, Texas A&M University Health Science Center, Bryan, TX, United States

**Keywords:** PTSD, animal models, pre-clinical/preclinical, traumatic stress, comorbidity

This editorial outlines the *Research Topic: Pre-clinical Models of PTSD* that includes 16 publications: 11 original research articles, 2 reviews, 2 mini-reviews, and a perspective that all integrate rodent models of posttraumatic stress disorder (PTSD) in the conceptual framework of the publications. By using rodent models of traumatic stress exposure and/or measuring PTSD-specific physiologic characteristic and PTSD-like behaviors, the articles explore both established and novel brain mechanisms implicated in PTSD. The manuscripts span neurochemical and anatomical levels, sex-dependent differences, relationships to comorbid conditions, and new rodent models of PTSD-like effects.

Similarly to other brain based pathologies, pre-clinical models of PTSD are critical for the ability to mechanistically explore the effects that trauma have on brain structure and function, yielding considerable insight into the neurobiological processes that likely underlie PTSD phenotypes. This *Research Topic* contains manuscripts that continue this exploration, including studies that focus on brain catecholamine signaling in well-characterized and novel neurocircuitry implicated in PTSD. Papers by Chaby et al., Deslauriers et al., and Liu et al. examine aspects of catecholamine signaling as a key system relevant to the expression of PTSD-like behaviors with potential relevance to PTSD treatment. Manuscripts by Piggott et al. and Miles and Maren, and others investigate specific brain regions that likely play an important role in PTSD, including prefrontal cortex and amygdala, as well as brain regions, that have been less studied in this context, namely the striatum and bed nucleus of the stria terminalis. This subgroup of papers that explore involvement of brain mechanisms, often linked with reward, provides conceptual bridge for the understanding of the highly prevalent association of PTSD with substance use disorders, a comorbidity that is reviewed by Gisquet-Verrier and Le Dorze.

Beyond direct mechanistic studies, PTSD-related animal models provide a means to study additional risk and protective factors that can modify the development of psychopathology following exposure to traumatic stress. This *Research Topic* includes a number of studies on the mitigating role of sex and developmental factors in expression of PTSD following trauma. For example, Asok et al. show that fear generalization is sex-dependent, Colom-Lapetina et al. illustrate a sex-dependent diversity in behavioral response to trauma, and Nahvi et al. provide evidence that females show anxiety- and depression-like behaviors akin to males, but have a unique neuro-mechanism underlying these behaviors. Interestingly, Chen et al. using two animal models of PTSD report that while fear extinction learning deficits manifest when trauma occurs in adulthood, adolescent animals might be resilient to similar trauma. Together these studies contribute to the growing body of work addressing the need to understand factors, such as sex and age that contribute to PTSD, highlighting the need to use multiple models of PTSD to define specific aspects or uncover unique characteristics associated with traumatic stress exposure.

Historical evidence shows that with new models new candidate mechanisms come into the investigative focus. The *Pre-clinical Models of PTSD Research Topic* embraces several manuscripts that involve new models and/or investigate new mechanisms potentially relevant to PTSD. For instance, Paredes and Morilak review the use of extinction learning as a model of exposure therapy in rodents, Conoscenti and Fanselow discuss key aspects that differentiate animal models in the stress literature, and Pinna uses a novel social-isolation model to determine if a biomarker, allopregnanolone in this case, can be identified for PTSD. Furthermore, this *Research Topic* delves into novel mechanisms, such as those explored by Moshfegh et al. involving inflammatory response to stress, or the effects of fear extinction learning on conditioned cardiovascular response by Swiercz et al. It also includes papers that investigate neurotherapeutics for PTSD, such as a study by Shallcross et al. that studied cannabidiol and a mGlu5 positive allosteric modulator as potentially useful agents with unique effects on stress and fear-related behaviors. These novel models and mechanisms continue to expand our understanding of PTSD and emphasizing the importance of scientific and conceptual diversity in PTSD research.

The collection of manuscripts in *Pre-clinical Models of PTSD* focus on the use of animal models to advance understanding of PTSD-related pathophysiology, including mechanisms targeting aspects of the brain, the body, and individual behavior. Moreover, factors affecting vulnerability and resilience, comorbidities, and treatment outcomes are considered in this *Research Topic*. Finally, while recognizing that PTSD is a uniquely human disorder, the use of appropriate animal models of PTSD and traumatic stress exposure and measurement of defining characteristics of PTSD remain critical components of the research endeavor to understand the complex neurobiology of PTSD.

## Author Contributions

All authors listed have made a substantial, direct and intellectual contribution to the work, and approved it for publication.

### Conflict of Interest

The authors declare that the research was conducted in the absence of any commercial or financial relationships that could be construed as a potential conflict of interest.

